# Multi-task learning with a natural metric for quantitative structure activity relationship learning

**DOI:** 10.1186/s13321-019-0392-1

**Published:** 2019-11-12

**Authors:** Noureddin Sadawi, Ivan Olier, Joaquin Vanschoren, Jan N. van Rijn, Jeremy Besnard, Richard Bickerton, Crina Grosan, Larisa Soldatova, Ross D. King

**Affiliations:** 1 Department of Medicine, Imperial College London, London, UK; 2 Department of Applied Mathematics, Liverpool John Moores University, Liverpool, UK; 3 Eindhoven University of Technology, Eindhoven, The Netherlands; 4 Leiden University, Leiden, The Netherlands; 5 University of Dundee, Dundee, Dundee, UK; 6 Ex Scientia Ltd, Dundee, UK; 7 Brunel University London, London, UK; 8 Goldsmiths, University of London, London, UK; 9 University of Manchester, Manchester, UK

**Keywords:** Multi-task learning, Quantitative structure activity relationship, Sequence-based similarity, Random forest

## Abstract

The goal of quantitative structure activity relationship (QSAR) learning is to learn a function that, given the structure of a small molecule (a potential drug), outputs the predicted activity of the compound. We employed multi-task learning (MTL) to exploit commonalities in drug targets and assays. We used datasets containing curated records about the activity of specific compounds on drug targets provided by ChEMBL. Totally, 1091 assays have been analysed. As a baseline, a single task learning approach that trains random forest to predict drug activity for each drug target individually was considered. We then carried out feature-based and instance-based MTL to predict drug activities. We introduced a natural metric of evolutionary distance between drug targets as a measure of tasks relatedness. Instance-based MTL significantly outperformed both, feature-based MTL and the base learner, on 741 drug targets out of 1091. Feature-based MTL won on 179 occasions and the base learner performed best on 171 drug targets. We conclude that MTL QSAR is improved by incorporating the evolutionary distance between targets. These results indicate that QSAR learning can be performed effectively, even if little data is available for specific drug targets, by leveraging what is known about similar drug targets.

## Introduction and problem specification

Rich Caruana in his widely cited paper defined multi-task learning (MTL) (see the list of Abbreviations below) as *“an approach to inductive transfer that improves generalization by using the domain information contained in the training signals of related tasks as an inductive bias. It does this by learning tasks in parallel while using a shared representation; what is learned for each task can help other tasks be learned better”* [1]. A more formal definition of MTL is given in  [2]:

### **Definition**

*(MTL)*: Given *m* learning tasks

$$\begin{aligned} \left\{ T_i \right\} _{i=1}^{i=m} \end{aligned}$$

where all the tasks or a subset of them are related but not identical, MTL aims to help improve the learning of a model for $T_i$ by using the knowledge contained in the *m* tasks.

There are three aspects of the task relatedness: feature, parameter, and instance; and correspondingly---three types of MTL [2]:

1. *Feature-based MTL models* assume that different tasks share identical or similar feature representations, which can be a subset or a transformation of the original features.
2. *Parameter-based MTL models* aim to encode the task relatedness into the learning model via the regularization or prior on model parameters.
3. *Instance-based MTL models* propose to use data instances from all the tasks to construct a learner for each task via instance weighting.

In recent years, MTL has been an active research area within the machine learning community and beyond. Instance-based MTL is among the most popular approaches because it often yields improved predictive performance  [3, 4]. The intuition is that by combining training data across multiple related tasks, each task benefits from the related information in other tasks, resulting in higher accuracy learning [5]. In other words, model generalization for individual tasks can be enhanced by sharing representations among tasks that are related.

MTL is considered as a sub-area of transfer learning [6]. The idea of transfer learning is to extract knowledge from one or more *source* domains, and reuse this knowledge in a *target* domain where data is scarce, with the aim of building better performing learning models in the target domain [7].

In this work we apply instance-based and feature-based MTL for the problem of predicting quantitative structure activity relationship (QSAR). The goal of QSAR learning is to learn a function that, given the structure of a small molecule (a potential drug), outputs the predicted activity of the compound against an assay (a test that predicts the potential of the compound being a drug) [8].

QSAR modelling has come a long way since its establishment in the early 1960s [9]. Although many drug targets are well studied and analyzed, a considerable number of them is still not, meaning that the quantity of labelled data for such targets is scarce (i.e. the number of chemical compounds with known bioactivity against these targets is small). Therefore, this leads to poor quality QSAR models which hampers understanding of these drug targets. Accurate predictive QSAR models are key for the discovery of new bioactive chemical compounds [10].

A single task $T_i$ is a task of predicting an activity $A_i$ given a QSAR dataset of molecular structures (see Table 1 for a typical example of QSAR dataset and “Data” section for further explanations). MTL is a suitable approach for the considered problem because:

- Different QSAR learning tasks share identical feature representations. For example, one of the most widely-used representations is fingerprints (see “Data” section for further detail).
- There are publicly available datasets for many QSAR tasks, and these data instances can be used to construct a learner for each task via instance weighting (see “Methods” section for further detail).
- It is also possible to apply parameter-based MTL, because there are available parametric QSAR models, although this is outside of the scope of this paper.

The application of MTL for QSAR learning in particular is beneficial because a considerable number of drug targets remains poorly studied and the quantity of labelled data for such targets is scarce. It is costly to obtain labeled data and this limits opportunities for constructing high-quality QSAR models and advancing understanding of these drug targets. In this paper we report the results of the use of existing data from related drug targets, where labeled data is aplenty, to predict activities for the drug targets where data is scarce. Our method is to use MTL where we exploit the drug target relatedness through the incorporation of the natural evolutionary metric. Specifically, in this paper we test the following two hypotheses:

1. MTL can improve on standard QSAR learning through the use of related targets.
2. MTL QSAR can be improved by incorporating the evolutionary distance of targets.

**Table 1:** A typical QSAR dataset

MOL_ID	FP_1	FP_2	...	FP_n	Activity
ID_1	1	0	...	1	6.351
ID_2	0	1	...	0	7.534
...	...	...	...	...	...
ID_22	1	1	...	1	8.001
ID_23	0	1	...	0	6.239

## Related work

### Multi-task learning

MTL has been used in many areas. For example, Chen et al. employed MTL to learn a common feature space from multiple related tasks and applied it for web page categorization [11]. Bickel et al. applied MTL for HIV therapy screening data with the focus on assigning weights to instances from multiple tasks so that tasks can be learned jointly even if data for different tasks have arbitrary different distributions [12]. Bickel et al. introduced a new MTL method for weighting groups in tree guided group-lasso regression and applied it for the analysis of genotype and gene expression data [13].

Zhang et al. reported on a multi-modal multi-task (M3T) method for simultaneously predicting multiple outcomes for multi-modal data [2]. The method is based on selecting common relevant features, applying kernel based data fusion and then applying multi-outcome support vector regression. Experiments were performed to jointly predict clinical scores in Alzheimer’s disease.

### Deep learning

Deep learning has gained significant attention over the last years and there are attempts to employ it for MTL. For example, deep relationship networks (DRN) were proposed to estimate the relationships between tasks in the area of computer vision [14]. In natural language processing (NLP), MTL was used with deep learning for identifying better hierarchies for tasks to improve performance [15].

### Task relatedness

A number of approaches have been reported in the literature for the specification of task similarity, an important element of MTL. One common approach is to build models on the individual tasks, and then to learn a common prior over the trained model parameters. For instance, this prior can be inferred using Dirichlet processes [16], matrix-variate normal distributions [17], or a maximum likelihood procedure [18]. Clustered multi-task learning (CMTL) preforms clustering of tasks into groups prior to applying MTL. This clustering can be done both on the task level [3, 19, 20] and on the level of shared feature representations among tasks [21–24].

Discovering highly important marker genes was the main focus of the work reported in [25] where the aim was to identify a shared gene subspace across different gene expression datasets using MTL. Zhou et al. modeled disease progression by considering predictions at different time points as different tasks and transform the problem into MTL  [26]. The relatedness between tasks was obtained by using a temporal group Lasso regularizer.

Taxonomy-based MTL was used to conduct biological sequence classification for the purpose of predicting the splice sites in various drug targets [27]. In this approach, the relatedness of tasks was defined by a phylogenic tree based structure and learning was performed at different levels of the tree. Furthermore, taxonomy- and graph-based transfer learning and MTL were used to predict the binding of the major histocompatibility complex (MHC)-I [28]. Although task relatedness can be derived from the hierarchy, the authors report an interesting approach to quantify this relatedness using multi-kernel SVMs. Also, a two step MTL approach was employed for the prediction of small interfering RNA (siRNA) efficacy [29]. In the first step, shared-task representations are learned, and in the second step, these representations are fed into a regressor to model each task.

A methodology that employs sequence based distance is described in [30]. In this approach an attempt was made to predict the similarity in binding profile between any pair of kinases from the human kinome. A binding profile was built for each kinase and it was used to compute pairwise similarity between kinases. This similarity was compared with the sequence based distance in order to check whether there is any correlation between the two. The difference between our approach and this approach is that we use the pairwise sequence based similarity between drug targets as input features to the classifier. Also, unlike our work, this method does not allow predicting the activity of individual molecules on drug targets.

### Multi-task learning for QSAR learning

MTL employing neural networks is reported in [31]. Multi-target predictions were made for a total of 19 assays at the same time. Although training is conducted by combining data from multiple assays, this method does not take advantage of the task relatedness. The QSAR problem is considered as a classification problem (i.e. whether a compound is active or inactive in a certain assay). This is different from our approach where we treat QSAR as a regression problem, and we work with a considerably larger number of assays (1091 assays).

Work applying MTL in QSAR learning includes applications in sequence biology [28] using a graph-based regularization method [3, 32] based on SVM [33]. Experiments were performed on data from the human kinome, and the relatedness between tasks was extracted from the taxonomy of kinase targets. A distance matrix was derived from the taxonomy by considering the distance between two taxa as the weight of the shortest path between them in the taxonomy [34]. This matrix was then transformed into a similarity matrix and the values were used to perform MTL. This measure of similarity is different from the homology used in our work, and it is less biologically meaningful. Ning et al. used SVM-based MTL approach to learn a classification model for a drug target together with other related drug targets, where compound- and target-specific kernel functions were used to capture intrinsic commonalities [35].

One of the key QSAR studies that employed MTL as well as transfer learning was reported in [36]. In addition to MTL, the approach uses feature nets (FN) to construct neural network and partial least squares (PLS) models for the modeling of 11 types of tissue-air partition coefficients. A total of 56 and 50 models for H/tissue and R/tissue respectively were obtained in the experiments which demonstrated the usefulness of MTL and transfer learning in general. The reported approaches showed that these techniques are specially useful when data is scarce. Our approache is different in multiple ways. We performed experiments on a much larger scale. Also, the authors did not evaluate traditional machine learning methods to select the best performing ones for STL. In particular, random forest (RF) was not considered [36]. This could be due to the used descriptors: we worked with fingerprints whereas they worked with some physicochemical properties as well as ISIDA descriptors [37]. In addition, our results are more statistically significant.

A recent approach, that reports significant improvements over traditional baseline machine learning approaches, applied massively multi-task neural networks for drug discovery [38]. In this work, an attempt was made to use deep learning to provide a framework for sharing information across a large number of datasets. The end goal was to classify compounds as either active or inactive.

Another approach that employs deep neural networks (DNN) is the work presented in [39] which tried to not only demonstrate that multi-task DNNs work in QSAR but also to explain why this is the case. The authors report that some form of signal transfer takes place between structurally similar molecules during the training process, and this can lead to better performance when molecule activities are correlated. A recent review of applications and challenges of MTL and transfer learning in QSAR can be found in [40].

### Advantages of the proposed approach

The proposed approach has the following advantages compared with the previous MTL work:

1. The QSAR learning problem is considered as a regression problem. This is more natural as finding the best threshold value to determine whether a specific compound is *active* or *inactive* is problematic and often results in loss of information.
2. We employ RF as the base learner. We showed in a previous study that RF outperforms other learners on QSAR data in the majority of scenarios [41].
3. We employ the functional-class fingerprints (FCFP) method to represent molecular structures. We have empirically found them to generally be the most successful QSAR prediction representation. We have done this by performing tests and comparisons using thousands of datasets and several learners [41].
4. One of the contributions of our work is the use of the drug target similarities in an MTL setting. The majority of existing MTL approaches focus on learning the task similarities, whereas in our case, we exploit the sequence based similarities and incorporate them in our experiments. There are often commonalities in QSAR assays as the target proteins may be evolutionary related. We took advantage of this and used protein sequence similarity values as our task similarities. This enables the inference of a natural metric of evolutionary distance between the drug targets.

In this paper we introduce an intuitive, simple and effective method of learning QSARs jointly. We test whether our MTL method can improve on standard QSAR learning through the use of related targets, and evaluate whether QSAR MTL can be improved by incorporating the evolutionary distance between targets. Our method is based on the classification of drug targets into families and the use of sequence similarity values between those drug targets [42].

## Data

We obtained drug activity data from the publicly available database ChEMBL containing curated records about the activity of specific compounds (drugs, small molecules) on drug targets (proteins) [43]. Activities in ChEMBL, e.g. potency and affinity endpoints, are recorded as real values (i.e. IC50, EC50, Ki, Kd and their equivalents).

In this study we used IC50 values, inhibitory drug concentrations at 50%. IC50 value states the concentration of the drug compound that is required to block or inhibit 50% of the proteins. This response data has been normalised by taking the negative log of the drug concentrations that inhibited 50% of a target (pXC50):

$$\begin{aligned} pXC50=-log_{10}IC50 \end{aligned}$$

The pXC50 provides a continuous scale of 1–12 where a compound of the value 1 is the least potent inhibitor and requires a large concentration of the drug to achieve 50% inhibition and 12 is the most potent inhibitor requiring a very low concentration to achieve 50% inhibition. In a small proportion of cases, where multiple activities have been reported for a particular compound-target pair, a consensus value was selected as the median of those activities falling in the modal log unit. Therefore, the unit of activity we are referring to is the *pseudo-pIC50*. This is a similar procedure to what was used in the AEROPATH target database project [44].

ChEMBL provides two ways of categorizing drug targets: a 6-level hierarchical *classification* of protein families, and a *grouping* of drug targets by their *preferred names*. In this paper, we perform MTL on the level of both groups and classes.

### Drug target classes

In the 6-level hierarchy, the ChEMBL database curators have classified protein targets into a manually curated family hierarchy according to nomenclature commonly used by drug discovery scientists, e.g. a ligand-based classification of G-protein-coupled receptors, and a division of enzymes into proteases/kinases/phosphatases. The version of the hierarchy used in this study is ChEMBL20, and it comprises of 6 levels, with Level 1 (L1) being the broadest class and Level 6 (L6)—the most specific. For example, the protein “tyrosine-protein kinase Srms” is classified as follows: enzyme (L1), kinase (L2), protein kinase (L3), TK protein kinase group (L4), tyrosine protein kinase Src family (L5), tyrosine protein kinase Srm (L6). Different classes in L1 are not evolutionary related to one another, whereas members of classes in L3 and below share common evolutionary origins for the most part. The picture is mixed for L2. The hierarchy is not fully populated, with the greatest emphasis being placed on the target families of highest pharmaceutical interest, and the different levels of the hierarchy are not defined by rigorous criteria. However, the hierarchical classification provides a useful means of grouping related targets at different levels of granularity.

### Drug target groupings

The method using preferred names is based on the practice that individual proteins can be described by a range of different identifiers and textual descriptions across the various data resources. The ChEMBL curators have assigned each protein target a preferred name in a robust and consistent manner, independent of the various adopted names and synonyms used elsewhere. The detailed manual annotation of canonical target names means that, for the most part, orthologous proteins from related species are described consistently, allowing the most related proteins to be grouped together. In the preferred name groupings, we obtained 468 drug target groups. The minimum number of drug targets in a group is two, and the maximum number of drug targets is 21 for the dihydrofolate reductase group (DHFR).

### Drug targets similarity

In our approach we employ evolutionary relatedness of drug targets as a similarity metric between drug targets within each drug target group or class. Drug targets similarity is based on the similarity of their amino-acid sequences. Sequence alignment is a method to detect regions of similarity among sequences [45]. There are two types of alignment: global and local. In global alignment the full lengths of sequences are aligned, whereas in local alignment, only parts of the sequences are aligned. Often, the Needleman–Wunsch algorithm [46] is used for performing global sequence alignment and the Smith–Waterman algorithm [47] is used to carry out local sequence alignment.

To obtain a metric for the similarity of protein targets we pairwise aligned their sequences using the Smith–Waterman algorithm and measured amino-acid residue similarity. We used the full sequence as the active sites are not easily labeled. Using active sites might further improve the results, but in this study we opted for the simplest option.

In more detail, given a pairwise sequence alignment of related protein sequences, it is common practice to quote the value of percentage sequence identity (PID) as a simple measure of evolutionary relatedness. This gives us a metric of evolutionary distance that ranges between zero and one; with numbers closer to one indicating more related drug targets. There is no universally accepted standard method to calculate PID [48]. In this work *PID*1 method (the default setting in BioStrings [49]) was used:

$$\begin{aligned} PID1 = 100 * \frac{identical\ positions}{aligned\ positions\ +\ internal\ gap\ positions} \end{aligned}$$

## Methods

Based on our previous extensive comparative study of conventional learners [41] showing that RF [50] outperforms other learners for the majority of QSAR problems, we decided to employ RF with 100 trees as our base learner, and evaluate its performance with tenfold cross-validation. We have chosen to use the Root Mean Squared Error (RMSE) [51] as the evaluation measure because we are predicting a real value number (the pseudo-pIC50).

We used the FCFP 1024-bit molecular fingerprints to represent molecules. Molecular fingerprints encode the structure of a chemical compound as a series of binary digits that indicate the presence or absence of particular substructures in the molecule [52]. For example, if a molecule contains a benzyl ring, the corresponding bit in the fingerprint will be 1, and if not—0. This molecule representation was selected because it is highly reliable for QSAR problems. More details can be found in our previous work [41] where we carried out an extensive comparison between several representations such as different kinds of fingerprints and descriptors.

In this work we performed all experiments using WEKA 3.7.11 machine learning library [53]. The implementation was done in Java utilising WEKA API as the basis for building our algorithms and running experiments.

### Single task learning

As a baseline, we include a single task learning (STL) approach that trains RF to predict drug activity for each drug target individually. Table 1 shows an example QSAR dataset, consisting of chemical compounds, their fingerprints and activity values. We will refer to this setting as STL.

### Feature-based MTL

In the feature-based MTL approach we aim to learn all drug targets for a particular protein target group (e.g. DHFR) or class (e.g. AMPA receptor) simultaneously. This was done by concatenating all the datasets of the same group or class, and adding an extra *indicator attribute*. As shown in Table 2, the *Target ID* attribute, *TID* for short, indicates which drug target, or species, the example came from (e.g. *P. falciparum*). As dataset entries (i.e. examples) are molecules, we give each molecule a unique identifier (*MOL_ID*). This helps to keep track of molecules even if the same molecule appears in more than one dataset.

**Table 2:** An input dataset for feature-based MTL

MOL_ID	TID	FP_1	FP_2	...	Activity
ID_1	7	1	0	...	6.351
ID_2	7	0	1	...	7.534
...	...	...	...	...	...
ID_111	95	1	1	...	8.001
ID_112	95	0	1	...	6.239

Algorithm 1 shows the pseudocode of feature-based MTL. We ran RF (with 100 trees) on the concatenated dataset and performed tenfold cross-validation to obtain an estimate of the performance. We used stratified sampling based on the TID attribute for cross-validation [54]. Such sampling procedure ensures that, when randomly selecting a sample from the population, the proportion of each group in the sample is the same as in the original population. Although this is usually done in the context of classification problems with imbalanced classes, we employ it here to ensure that our per-fold performance estimates are based on the actual distribution of drug targets in the original data. We evaluate the performance (RMSE) of our MTL approach for each TID separately by filtering out the predictions for that specific TID in each test set.

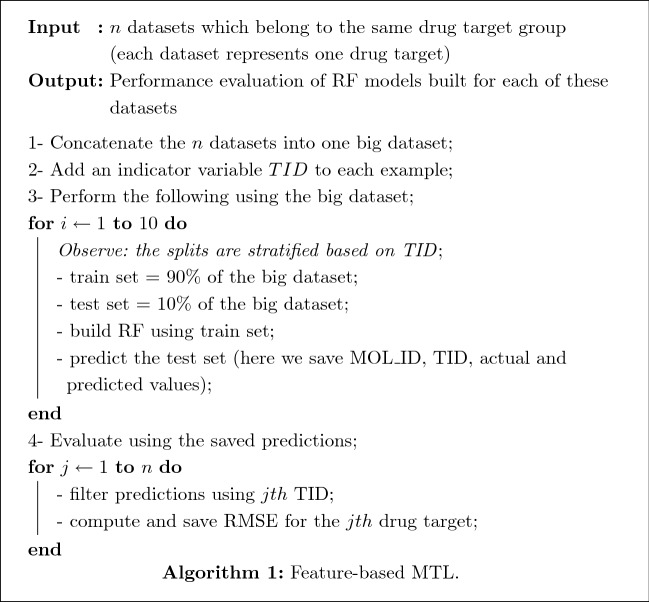



By the end of the cross-validation, we obtained a list of all molecules and their respective TIDs, and their actual and predicted activity values (see Table 3). In order to examine the performance of RF on any particular drug target, we select instances that are from that particular target’s dataset by filtering TIDs. This gives the actual and predicted activity values for that particular dataset, and it is straightforward to compute RMSEs (see “Results and discussion” section). The same procedure is used for the evaluation of the performance of instance-based MTL.

**Table 3:** An output table for feature-based MTL

FOLD	MOL_ID	TID	Activity	Prediction
1	ID_1	7	6.351	6.011
1	ID_2	7	7.534	7.681
...	...	...	...	...
10	ID_111	95	8.001	7.764
10	ID_112	95	6.239	6.401

### Instance-based MTL

In the instance-based MTL we made use of the quantitative similarity between drug targets described in “Drug targets similarity” section. To represent this information, we added *n* extra attributes that consist of the similarity values to the other species (*n* is the number of drug targets in each drug target group or class). As Table 4 shows, the attribute $SimToTID\_7$ gives the similarity value between drug target with TID 7 and all other drug targets in this concatenated dataset. For examples which belong to TID 7, this value will be 1.

**Table 4:** A dataset for instance-based MTL

MOL_ID	TID	SimToTID_7	...	SimToTID_95	FP_1	FP_2	...	Activity
ID_1	7	1	...	0.584	1	0	...	6.351
ID_2	7	1	...	0.584	0	1	...	7.534
...	...	...	...	...	...	...	...	...
ID_111	95	0.584	...	1	1	1	...	8.001
ID_112	95	0.584	...	1	1	1	...	6.239

Algorithm 2 shows the pseudocode of instance-based MTL. We again used RF with 100 trees on the concatenated dataset, which now contains the similarity values. As in feature-based MTL, we used stratified tenfold cross-validation training based on the TID to evaluate the performance of instance-based MTL approach, and compute the RMSE for each TID individually.

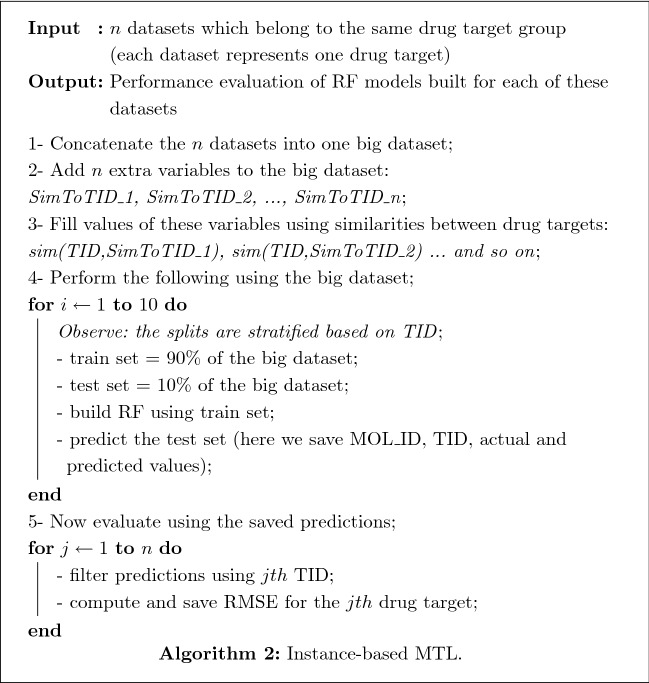



## Results and discussion

To evaluate the performance of our MTL approach, we performed MTL on the level of all groups and classes of drug targets, building a model simultaneously for all drug targets within that group or class. We only considered groups or classes that have more than one drug target, because otherwise there would be no difference with STL, and only included drug targets for which the minimum size of their dataset was 10, because we employ tenfold cross-validation. In other words, each dataset must contain at least 10 compounds with their corresponding activity against that drug target.

We compared the three settings discussed in “Methods” section by running MTL on all drug classes and groups, obtaining a list of drug targets with their corresponding RMSE values for STL, feature-based and instance-based MTLs. Finally, we counted the number of cases where each setting had lowest RMSE.

To examine the distribution of RMSE values for each setting we drew histograms, ran Shapiro–Wilk tests [55], generated Q–Q plots, and concluded that these values do not follow a normal distribution. Hence, we applied the non-parametric Wilcoxon Signed-ranks test to examine whether or not the difference between these values is statistically significant. For each experiment, we show the results of three different Wilcoxon Signed-ranks tests to pairwise compare the RMSE performance of the three settings. The following subsections show the details of our experiments using ChEMBL’s 6-level hierarchical classification and its grouping by preferred names.

### Using ChEMBL’s class levels

We previously described ChEMBL’s 6-level hierarchical protein family classification which starts with L1 (most generic class) to L6 (most specific class). Table 5 displays the number of classes we obtained at each level. Note that Table 5 shows the number of classes at each level in the hierarchy explained in “Drug target classes” section, and this is different from the number of groups in the preferred named grouping explained in “Drug target groupings” section.

**Table 5:** ChEMBL’s 6-level protein family classification

Level	No of classes
L1	13
L2	24
L3	46
L4	111
L5	180
L6	50

Broad classes such as enzyme and membrane receptors can be found at L1, whereas as we traverse down the hierarchy, we can find more specific classes such as antiporter and protein kinase at L3 and amine and motilin receptor at L5. It is reasonable to assume that more specific classes are more evolutionarily related. L5 has more classes than any level, i.e. 180, as shown in Table 5. Over the total of 1091 drug targets (corresponding to 1091 assays we run experiments for), we expect that a grouping at L5 would yield sets of targets which are closely related. Therefore, we present our experimental results using this level.

**Fig. 1:**
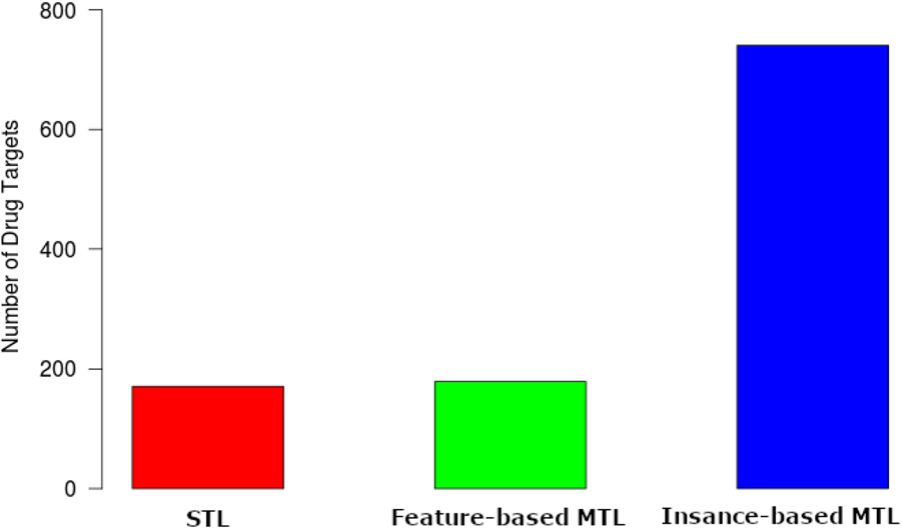
The number of drug targets each method scores the lowest RMSE value

**Fig. 2:**
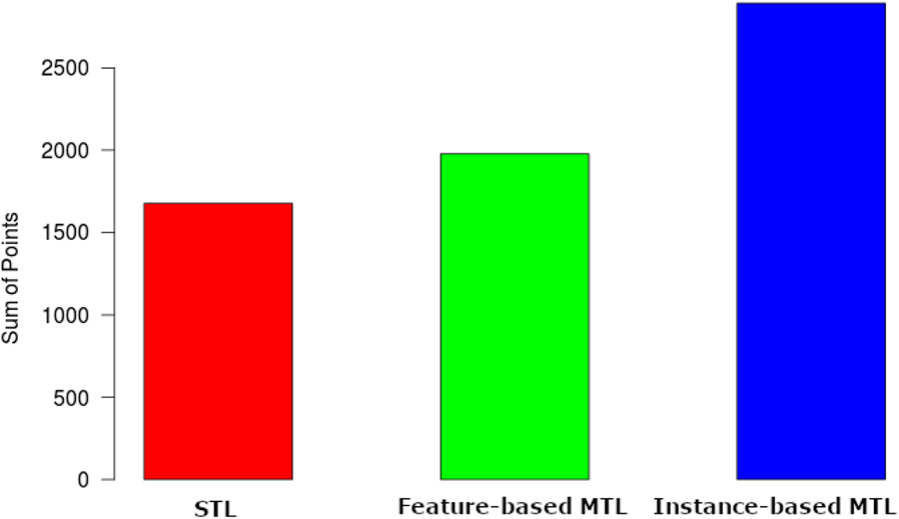
Feature-based and instance-based MTL compared with STL (ranked from 3 to 1) using L5 classes

Table 6 shows a simple sign test where we count how many times the RMSE value for each algorithm is less than the other. The +ve column indicates how many times the RMSE for the first setting is less than the second setting while the −ve column indicates how many times the RMSE for the second setting is less than the first setting. This shows that, for instance, feature-based MTL outperforms STL in 686 of the cases. Counting the number of overall wins, shown in Fig. 1, yields that instance-based MTL outperforms both feature-based MTL and STL on 741 drug targets. Feature-based MTL won on 179 occasions and STL performed best on 171 occasions. The statistical significance of these results is shown in Table 7. Finally, Fig. 2 shows a point ranking where we award the best setting three points, the second best two points and the third best one point.

**Table 6:** Pair-wise sign test for the L5 results

Setting	# +ve	# −ve	# ties
Feature-based MTL vs STL	686	405	0
Instance-based MTL vs STL	911	180	0
Instance-based MTL vs feature-based MTL	891	200	0

**Table 7:** Pair-wise Wilcoxon signed-ranks test for L5 results (W is the test statistic)

Setting	W	p-value
STL vs feature-based MTL medians: 0.744 and 0.701	374646	1.609e−13
STL vs instance-based MTL medians: 0.744 and 0.633	535197	2.2e−16
Feature-based MTL vs instance-based MTL medians: 0.701 and 0.633	535673	2.2e−16

**Fig. 3:**
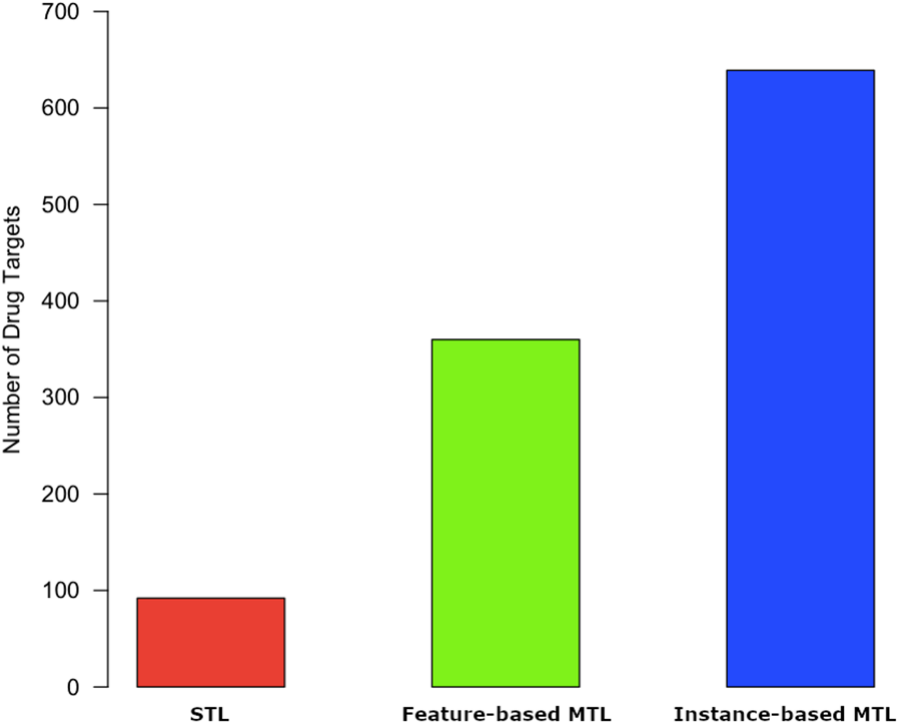
The number of drug targets each method scored the highest R-squared value

ChEMBL datasets are known to be imbalanced toward *active* compounds [56]; hence we have compared our methods using the coefficient of determination (also known as R-squared) [57]. Unlike RMSE where we are interested in the minimum value, when using R-squared we are interested in the highest value. This is because R-squared explains how good a model is. The value of R-squared normally ranges between 0 and 1, where 0 indicates a useless model and 1 indicates a perfect model. Our results are illustrated in Fig. 3. The figure shows how many drug targets each setting scores the highest R-squared on. Instance-based MTL outperforms both feature-based MTL and STL on 639 drug targets, feature-based MTL performs better than instance-based MTL and STL on 360 drug targets whereas STL performed best on 92 drug targets.

**Fig. 4:**
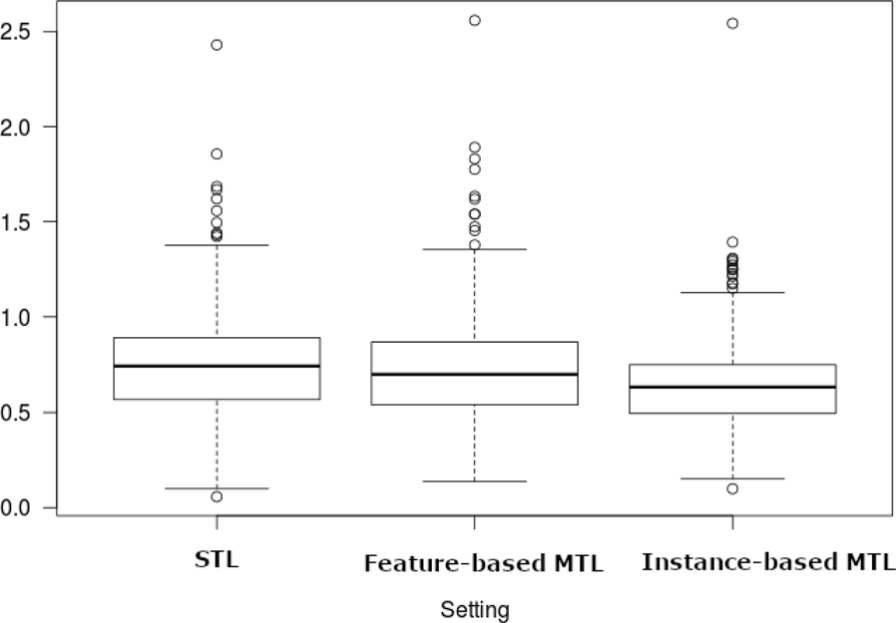
Boxplot of RMSE values for the three settings when applied to all L5 drug target classes

Table 7 shows the results of the pairwise Wilcoxon signed-rank tests. The null hypothesis is that the median change in RMSE values when we use our MTL methods is zero. As can be seen, feature-based MTL (median RMSE = 0.701) and instance-based MTL (Median RMSE = 0.633) both significantly outperformed STL (Median RMSE = 0.744). Moreover, instance-based MTL also significantly outperforms feature-based MTL. The difference in medians is further evident in the boxplot provided in Fig. 4.

For validation, we carried out a Y-randomisation procedure [58] on the feature-based MTL method. We repeated the same feature-based MTL procedure 1000 times using L5 target classes, and permuted the activity values each time. We then performed a sign test similar to that reported in Table 6, and the results demonstrate that RF (i.e. STL) performed significantly better than feature-based MTL in all runs. We have made all our source code and results available on the Github.

We also performed a randomisation procedure by shuffling the similarity values in the instance-based MTL approach. We have randomly selected 24 level 5 classes (the total number of selected drug targets is 120) and randomised their similarity values 1000 times. Each time we randomised, we run instance-based MTL and compute RSME for each drug target. Our results show that in 104 out of the 120 drug targets, the standard instance-based MTL approach performs better than when the similarity values are randomised (i.e. the RSME value for most drug targets when using the standard instance-based MTL approach is less than when randomising the similarity values). That is 86.67% of the randomly selected drug targets. This shows that the evolutionary data indeed significantly improves QSAR learners.

We have analysed the results of our work further by identifying what drug target classes benefited from the proposed MTL QSAR. We define a *fully benefited* class as an L5 class in which all drug targets have better results when using feature-based MTL as compared with STL or instance-based MTL as compared with feature-based MTL. On the other hand, we define a *no benefit* class as an L5 class in which none of the drug targets have better results when using feature-based MTL as compared with STL or instance-based MTL as compared with feature-based MTL. Our results show that there are 12 no benefit drug target classes, for examples Neurotensin receptor class with 4 drug targets and Cholecystokinin receptor class with 2 targets. We have also found that 40 drug target classes fully benefited from feature-based MTL. Examples are CMGC protein kinase RCK family with 2 drug targets and tyrosine protein kinase Trk family with 3 drug targets. On the other hand, only 9 drug target classes fall under the instance-based MTL no benefit class. Example classes are cytochrome P450 51A1 with 2 drug targets and aspartic protease A2A subfamily with 3 drug targets. Also, as many as 78 drug target classes fully benefited from instance-based MTL. Examples are tyrosine protein kinase EGFR family with 6 drug target and MCH receptor with 4 drug target. A list of all these drug target classes is provided on our Github repository.

Our results indicate that the size of no benefit classes are generally small with the highest number of drug targets in each class as 3. In addition, we have studied the similarity values amongst drug targets of fully benefited classes, and our analysis shows that instance-based MTL works better if there is a range of evolutionary distances in the class. In other words, if not all drug targets are very close or distant from each other.

### Using ChEMBL’s preferred name groups

Finally, we repeated our experiments using 468 drug target groups based on ChEMBL’s preferred name grouping (see “Data” section). For a more detailed analysis, we investigated the performance of the three settings on the largest drug target group we have, which is DHFR with 21 drug targets.

**Fig. 5:**
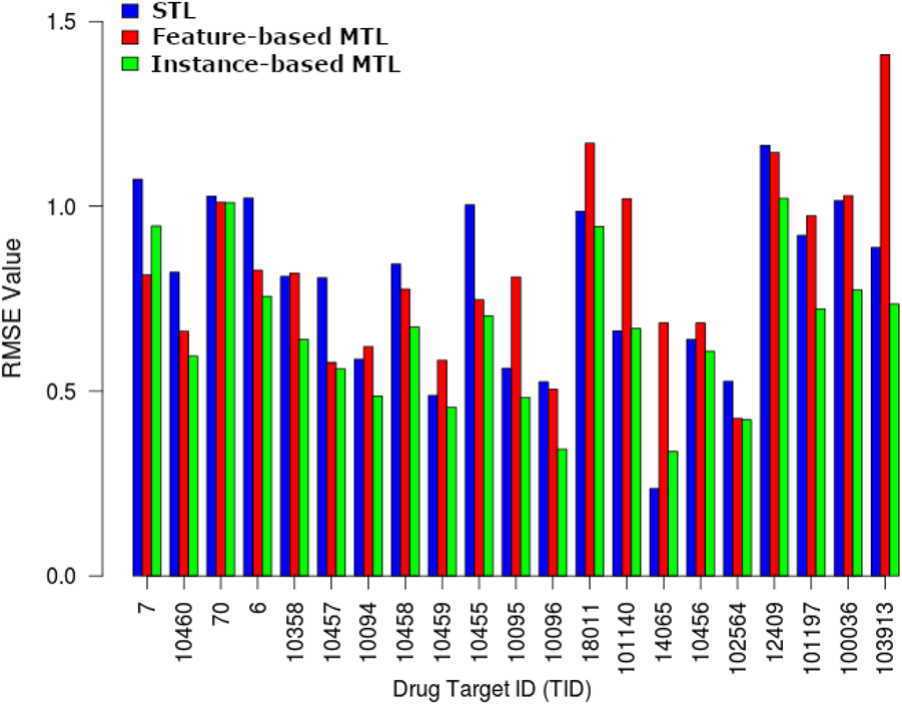
Barplot of RMSE values for 21 DHFR drug targets

Figure 5 shows a barplot of the RMSE values for the three settings on each of the 21 drug targets in the DHFR group. Instance-based MTL outperformed both feature-based MTL and STL in 18 drug targets and was never the third best. The STL was the best performer for only two drug targets whereas feature-based MTL won on only one drug target.

**Fig. 6:**
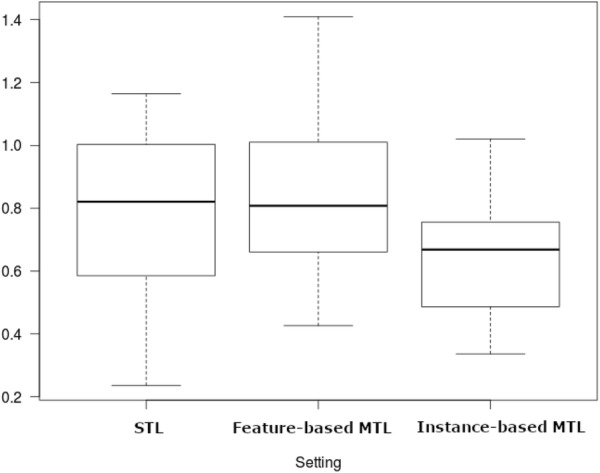
Boxplot of RMSE values for the three settings when applied to 21 DHFR drug targets

Table 8 shows the results of the pairwise Wilcoxon signed-rank test. The null hypothesis is that the median change in RMSE values when we use our MTL methods is zero. As can be seen, for the specific DHFR group, there was no significant difference in the RMSE values for STL (Median RMSE = 0.821) and feature-based MTL (Median RMSE = 0.808). However, instance-based MTL (Median RMSE = 0.668) is significantly better than both STL and feature-based MTL. The difference in medians is evident in the boxplot provided in Fig. 6.

**Table 8:** Pair-wise Wilcoxon signed-ranks test for the 21 DHFR group results (W is the test statistic)

Setting	W	p-value
STL vs feature-based MTL medians: 0.821 and 0.808	108	0.8117
STL vs instance-based MTL medians: 0.821 and 0.668	222	3.147e−05
Feature-based MTL vs instance-based MTL medians: 0.808 and 0.668	220	5.245e−05

## Conclusions and future work

We have shown that MTL can significantly improve the performance of QSAR learning models, and thus can help to better predict the activity of drugs against specific drug targets. We predicted the activities of potential drugs against 1091 assays (i.e. 1091 drug targets) by grouping similar drug targets and training models on all targets within the same group simultaneously. Drug targets were grouped based on ChEMBL’s 6-level classification, as well as based on their preferred names.

The results show that MTL significantly outperformed learning QSAR models individually. Moreover, when incorporating a novel, natural similarity measure between drug targets based on their sequence alignment, and hence their evolutionary kinship, we can further significantly improve QSAR learning. These results indicate that QSAR learning can be performed effectively, even if little data is available for specific drug targets, by leveraging what is known about similar drug targets.

The QSAR datasets and experimental results are available on OpenML [59]. OpenML is an open source platform that facilitates discovering, sharing and reusing data, machine learning models and experiments. OpenML ensures that the submitted experiments are compliant with the W3C MLSchema [60], and therefore can be reproduced and reused in future work [61]. The Java source code for all reported experiments is freely available on Github [62]. The link also provides detailed information and a video demonstrating how the code can be run and how to analyse the results.

In future work, we plan to evaluate the performance of our methods without TID-based stratification. we intend to use the distance between drug targets instead of similarity values (distance = 1 − similarity) and use similarity, or distance, between datasets instead of drug targets and compare performance.

## Data Availability

All datasets and code are freely available at: https://github.com/nsadawi/MTL-QSAR.

## Abbreviations

QSAR: quantitative structure activity relationship
MTL: multi-task learning
DHFR: dihydrofolate reductase
MHC: histocompatibility complex
RNA: ribonucleic acid
DRN: deep relationship networks
FN: feature nets
PLS: partial least squares
NLP: natural language processing
CMTL: clustered multi-task learning
DNNs: deep neural networks
SVM: support vector machines
RF: random forest
FCFP: functional-class fingerprints
ChEMBL: Chemical Database at the European Molecular Biology Laboratory
PID: percentage sequence identity
TID: target identifier
MOL_ID: molecule identifier
RMSE: Root Mean Squared Error
STL: single task learning
AMPA receptor: $\alpha$-amino-3-hydroxy-5-methyl-4-isoxazolepropionic acid receptor
